# Association of Out-of-Pocket Spending With Insulin Adherence in Medicare Part D

**DOI:** 10.1001/jamanetworkopen.2020.33988

**Published:** 2021-01-26

**Authors:** Erin Trish, Katrina Kaiser, Geoffrey Joyce

**Affiliations:** 1 University of Southern California Schaeffer Center for Health Policy and Economics, Los Angeles, California; 2 University of Southern California, School of Pharmacy, Los Angeles, California

## Abstract

This cross-sectional study examines whether efforts to limit out-of-pocket spending for enrollees in nonsubsidized Medicare Part D plans are associated with insulin adherence rates among these patients.

## Introduction

About one-third of Medicare beneficiaries had diabetes in 2016, up from 18% in 2000.^1^ More than 3 million were taking insulin at a cost of $13.3 billion to Medicare and beneficiaries. Beneficiaries’ mean out-of-pocket spending on insulin has nearly doubled over the last decade, raising concerns about access to an essential medication.^1^ Reported cost-related underuse of insulin is high,^2^ leading experts to recommend policy changes to reduce out-of-pocket costs to limit negative health consequences and financial burden.^3,4^

In an effort to address these concerns, the Centers for Medicare & Medicaid Services (CMS) recently announced a voluntary program—the Senior Savings Model—to test the effect of limiting Part D beneficiaries’ out-of-pocket spending on insulin to no more than $35 per month starting in 2021.^5,6^

To assess the potential outcomes associated with this policy, we compared changes in basal insulin use before and after reaching the coverage gap, in which beneficiaries face increased cost-sharing, for 2 distinct groups of nonsubsidized Part D enrollees: (1) beneficiaries in individual plans whose cost sharing can vary dramatically across coverage phases; and (2) those enrolled through an employer group–waiver plan (EGWP) where cost sharing is low and fairly constant across coverage phases.

## Methods

Using a 100% sample of 2018 Medicare Part D claims (eAppendix 1 in the Supplement), we calculated mean out-of-pocket spending on basal insulin and adherence by plan type and benefit phase (eTable 1, eAppendix 2, and eAppendix 3 in the Supplement). To improve comparability across groups, we restricted our sample to previous users enrolled in enhanced Part D plans who did not receive low-income subsidies and ended the year in the coverage gap (48%) or catastrophic coverage (23%) (eTable 2 and eTable 3 in the Supplement). The University of Southern California institutional review board determined that the study met the criteria for coded private information or biological specimens and thus was exempt from informed consent requirements.

We calculated mean cost sharing per 30-day equivalent by dividing patient payments by number of 30-day equivalent claims (eAppendix 4 in the Supplement). We measured adherence as the percentage of days covered (PDC) (ie, days supplied divided by total days) (eAppendix 5 in the Supplement). Statistical analysis was performed using SAS Enterprise Guide version 7.1 (SAS Institute) from from January 2020 to November 2020.

## Results

Our analytic sample included 474 929 people who use basal insulin; 303 616 and 171 313 in individual and employer plans, respectively. Of people in the individual plans, the mean (SD) age was 73.1 (7.5) years, 52.6% (159 735) were men, and 81.2% (246 505) were White individuals. Demographic characteristics are similar across theindividual and employer plan groups. Among individual plan enrollees, mean (SD) cost sharing on basal insulin (per 30-day equivalent) was $50.57 ($44.40) in the initial coverage phase, $117.10 ($75.65) in the coverage gap, and $36.86 ($46.30) in catastrophic coverage (Figure). By contrast, mean (SD) out-of-pocket spending for employer plan enrollees was relatively low and consistent across the same 3 benefit phases: $32.73 ($30.21), $31.99 ($33.87), and $19.73 ($21.42), respectively.

**Figure.**
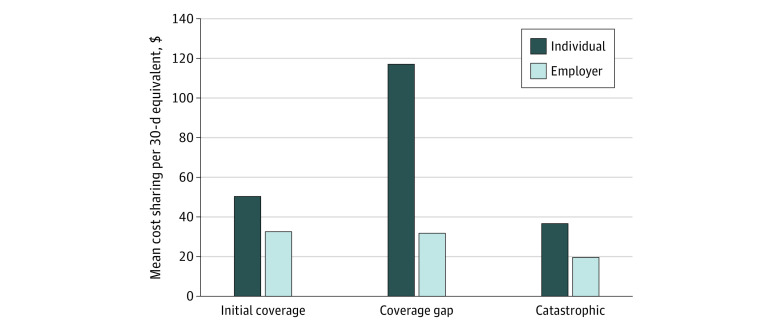
Mean Out-of-Pocket Spending on Basal Insulin per 30-Day Equivalent, by Part D Benefit Phase and Plan Type

Beneficiaries in individual plans who ended the year in the coverage gap reduced their use of insulin by a mean of 5.4 percentage points relative to their use in the initial coverage phase (mean PDC of 62.1% in coverage gap vs 67.5% in initial coverage) (Table). By contrast, employer plan enrollees’ insulin use increased in the coverage gap (PDC of 72.9% vs. 70.1%).

**Table.** Adherence to Basal Insulin by Part D Benefit Phase and Plan Type

Variable	Proportion of days covered, %		Difference: individual vs employer, percentage point
	Individual plan	Employer plan	
Users ending in coverage gap			
Initial coverage	67.5	70.1	−2.6
Coverage gap	62.1	72.9	−10.8
Catastrophic coverage	NA	NA	NA
Difference: coverage gap vs initial coverage, percentage point	−5.4	2.8	−8.2
Users ending in catastrophic coverage			
Initial coverage	73.6	75.8	−2.1
Coverage gap	73.6	78.2	−4.5
Catastrophic coverage	76.2	78.0	−1.8
Difference: coverage gap vs initial coverage, percentage point	0.0	2.4	−2.4
Users ending in coverage gap or catastrophic coverage			
Initial coverage	68.6	71.0	−2.4
Coverage gap	65.7	74.6	−8.9
Catastrophic coverage	76.2	78.0	−1.8
Difference: coverage gap vs initial coverage, percentage point	−2.8	3.6	−6.4

The pattern differs for those ending in catastrophic coverage. A person using insulin in an individual plan is likely to pay more upon reaching the coverage gap (Figure). However, each fill increases the likelihood of reaching catastrophic coverage, which lowers their expected out-of-pocket cost for future prescriptions that year (for insulin and all other drugs). We find that insulin use is largely unchanged across benefit phases for those ending in catastrophic coverage (Table).

## Discussion

In this study, mean out-of-pocket spending on insulin increased considerably in the coverage gap for individual plan enrollees, which was associated with a substantial reduction in adherence for some beneficiaries. Capping out-of-pocket spending on insulin at $35 per month, as required by the Senior Savings Model, will substantially reduce cost sharing for people who use insulin in the coverage gap and smooth patient liability across the year. Our study’s findings suggest that this may improve insulin adherence for some beneficiaries.

A limitation of our study is the uncertainty of claims-based measures of insulin adherence. Further research is needed to evaluate the effects on health outcomes.

## Notes

Supplement. **eAppendix 1.** Sample Construction **eTable 1.** List of Basal Insulins Included in Analysis, and Number of Claims (2018) **eTable 2.** Description of Sample Restrictions **eTable 3.** Year-End Benefit Phase By Plan Type **eAppendix 2.** Overview of Part D Benefit Phases **eAppendix 3.** Identification of Part D Benefit Phase on Claims **eAppendix 4.** Measuring Out-of-Pocket Spending **eAppendix 5.** Measuring Adherence **eReferences.**
