# Supplementary material for: Association of Out-of-Pocket Spending With Insulin Adherence in Medicare Part D
Source: JAMA Netw Open. 2021 Jan 26;4(1):e2033988. doi: 10.1001/jamanetworkopen.2020.33988 (PMC7838924; doi:10.1001/jamanetworkopen.2020.33988)
Supplement: Supplement. — eAppendix 1. Sample Construction eTable 1. List of Basal Insulins Included in Analysis, and Number of Claims (2018) eTable 2. Description of Sample Restrictions eTable 3. Year-End Benefit Phase By Plan Type eAppendix 2. Overview of Part D Benefit Phases eAppendix 3. Identification of Part D Benefit Phase on Claims eAppendix 4. Measuring Out-of-Pocket Spending eAppendix 5. Measuring Adherence eReferences. [file jamanetwopen-e2033988-s001.pdf]

Supplemental Online Content

Trish E, Kaiser K, Joyce G. Association of out-of-pocket spending with insulin adherence in Medicare Part D. *JAMA Netw Open*. 2021;4(1):e2033988. doi:10.1001/jamanetworkopen.2020.33988

eAppendix 1. Sample Construction
eTable 1. List of Basal Insulins Included in Analysis, and Number of Claims (2018)
eTable 2. Description of Sample Restrictions
eTable 3. Year-End Benefit Phase By Plan Type
eAppendix 2. Overview of Part D Benefit Phases
eAppendix 3. Identification of Part D Benefit Phase on Claims
eAppendix 4. Measuring Out-of-Pocket Spending
eAppendix 5. Measuring Adherence
eReferences

This supplemental material has been provided by the authors to give readers additional information about their work.

© 2021 Trish et al. *JAMA Network Open.*

eAppendix 1. Sample Construction

We analyzed a 100% sample of 2018 Part D enrollment and claims accessed through the CMS Virtual Research Data Center. The sample includes enrollees in stand-alone prescription drug plans (PDPs) and Medicare Advantage plans with Part D benefits (MA-PDs), including both non-employer (individual) and employer group waiver plans (EGWPs).

We restricted the sample to individuals who do not receive low-income subsidies (non-LIS) because most cost-sharing for LIS beneficiaries is paid by the federal government rather than the beneficiary.

We further restricted the sample to enrollees in enhanced plans to improve comparability between enrollees in individual and employer plans because the vast majority of employer plans are enhanced. Additionally, the CMS Senior Savings Model will apply only to enhanced plans.

Among this sample, we then identified insulin users according to the presence of one or more basal insulin claims in 2018 (the list of basal insulins is included in eTable 1). Basal insulins are generally longer-acting insulins that are typically taken once per day. We focus on basal insulins rather than short- or intermediate-acting insulins to improve our ability to measure adherence. Beneficiaries may also take additional (non-basal) insulins, but we do not include these claims in our measures of out-of-pocket spending nor adherence.

eTable 1: List of Basal Insulins Included in Analysis, and Number of Claims (2018)

Brand Name	Number of Claims
LANTUS SOLOSTAR	1,034,376
LEVEMIR FLEXTOUCH	508,644
LANTUS	432,853
TOUJEO SOLOSTAR	238,472
TRESIBA FLEXTOUCH U-200	119,443
LEVEMIR	105,467
TRESIBA FLEXTOUCH U-100	78,368
BASAGLAR KWIKPEN U-100	76,680
HUMULIN N	68,880
NOVOLIN N	37,833
HUMULIN N KWIKPEN	23,044
TOUJEO MAX SOLOSTAR	3,454

To avoid mismeasurement of adherence by new insulin users, we restrict our sample to basal insulin users who initiated their insulin prior to or on the first day of the year. To do so, we require that the individual had one or more basal insulin claims in 2017 or fill their first basal insulin claim of the year on January 1, 2018. We also exclude insulin users who die during 2018 to ensure that the study period is a full year for all included beneficiaries.

© 2021 Trish et al. *JAMA Network Open.*

After making these restrictions, our sample includes a total of 673,496 enrollees, with 454,200 (67%) individual plans enrollees and 219,296 (33%) employer-plan enrollees (eTable 2).

eTable 2: Description of Sample Restrictions

Restriction	Individual		Employer	
	Number Restricted	Remaining Total	Number Restricted	Remaining Total
Initial sample of basal insulin users	N/A	2,062,873	N/A	373,100
Keep only non-LIS beneficiaries	1,062,592	1,000,281	18,345	354,755
Keep only enhanced plan enrollees	250,035	750,246	301	354,454
Keep only PDP and MA-PD enrollees	9,824	740,422	3,283	351,171
Keep only insulin users who had a previous claim in 2017 (or on Jan 1, 2018) and did not die in 2018	286,222	454,200	131,875	219,296

Among this restricted sample of previous basal insulin users, about 29% ended the year in the initial coverage phase, 48% ended the year in the coverage gap, and 23% ended the year in catastrophic coverage (eTable 3). We restrict our analysis to the latter two groups of beneficiaries to focus on those beneficiaries who reach the coverage gap (and thus experience the associated increase in out-of-pocket spending, if applicable). The final analytic sample includes 303,616 and 171,313 basal insulin users enrolled in individual and employer plans, respectively.

eTable 3: Year-End Benefit Phase By Plan Type

Final Phase	Individual		Employer	
	Count	Percent	Count	Percent
Initial Coverage	150,584	33%	47,983	22%
Coverage Gap	204,377	45%	116,687	53%
Catastrophic Coverage	99,239	22%	54,626	25%
Total	454,200		219,296	

eAppendix 2. Overview of Part D Benefit Phases

The standard Medicare Part D benefit design includes multiple phases. In 2018, the standard benefit design for non-LIS beneficiaries included a $405 deductible, followed by an initial coverage phase, in which the beneficiary faced 25% coinsurance up to $3,750 in total drug spending, with the other 75% being paid by their plan. After that, they entered the coverage gap, in which they faced 35% coinsurance on branded drugs up to approximately $8,418 in total drug spending, with 50% being paid by the drug manufacturer and the remaining 15% being paid by the plan. After that, they entered catastrophic coverage, where they faced 5% coinsurance on all drug spending for the remainder of the year, with 80% being paid by the federal reinsurance program and the remaining 15% being paid by the plan.

© 2021 Trish et al. *JAMA Network Open.*

In practice, many plans do not follow the standard benefit design but instead use flat dollar copayments in the initial coverage phase such that actual beneficiary out-of-pocket spending on insulin is less than 25% of the drug's list price. Further, because our analytic sample is restricted to beneficiaries enrolled in enhanced plans, many plans provided more generous coverage of insulins in the initial coverage phase. However, even these enhanced plans tended to follow the 35% coinsurance in the coverage gap, because of the way that manufacturer-financed discounts are calculated. Because of Part D program rules, if a plan provided more generous coverage for branded drugs in the coverage gap, manufacturer liability would be calculated off the reduced patient liability rather than the list price of the drug. However, technicalities in Part D coverage essentially permit employer plans to provide more generous coverage in the coverage gap without foregoing these manufacturer-financed discounts. It is these different rules that drive the difference in cost-sharing between individual and employer plan enrollees that we observe in our analytic sample. Moreover, it is also these rules that will be amended as part of the Senior Savings Model to enable plans to provide more generous coverage in the coverage gap without foregoing the value of manufacturer-financed discounts.

eAppendix 3. Identification of Part D Benefit Phase on Claims

Part D claims for individual plans include a phase variable on each claim—we use this variable to identify the benefit phase for enrollees in individual plans. We categorize claims that span multiple phases (i.e., straddle claims) to the final benefit phase on the claim. In our analysis, figures reported for the initial coverage phase include claims in the deductible because many enhanced plans do not use a deductible and because we cannot separately identify deductible claims from initial coverage claims for employer plans.

Unlike claims for individual plans, claims for employer-based plans do not include a phase variable. We therefore impute the benefit phase for employer plans. As described above, in 2018 manufacturers were responsible for paying 50% of the list price of branded drugs for claims that occur in the coverage gap. In the Part D claims, this manufacturer-financed payment is captured in the "Gap Discount Amount (GAPDSCNT)" variable. All the basal insulins included in our analysis are branded drugs, and thus will include a positive amount for this variable when the claim falls in the coverage gap. A separate variable on each claim—"Gross Drug Cost Above Part D Out-of-Pocket Threshold (GDCA)"—captures the total spending for that claim that occurs in catastrophic coverage. Thus, we apply the following logic to claims for enrollees in employer-based plans: if both GAPDSCNT=0 & GDCA=0, then the claim is categorized as an initial coverage phase claim. If GAPDSCNT >0 & GDCA=0, then the claim is categorized as a coverage gap claim. If GDCA>0, then the claim is categorized as a catastrophic coverage claim. To reiterate, we cannot separate claims in the deductible from those in the initial coverage phase; thus, deductible phase claims are included in our initial coverage phase measures (for enrollees in both individual and employer-based plans).

© 2021 Trish et al. *JAMA Network Open.*

eAppendix 4. Measuring Out-of-Pocket Spending

We measure average out-of-pocket spending per 30-day equivalent by identifying the number of months supplied from the days supplied variable. 34 or fewer days supplied are one 30-day equivalent, 35 to 64 days supplied are two 30-day equivalents, and 65 days or more are three 30-day equivalents. We create the 30-day equivalent out-of-pocket amount for each claim by dividing the patient payment by this count of 30-day equivalents summing the total patient paid amount across all basal insulin claims, and calculate the mean and standard deviation across all claims. We restrict this to spending only on basal insulin claims (that is, we ignore spending by insulin users on other drugs, including other non-basal insulins). We calculate these measures by plan type and benefit phase in which the claim occurred.

eAppendix 5. Measuring Adherence

We measure adherence according to the percent of days covered, or the total basal insulin days supplied in a given benefit phase divided by the total number of days that the beneficiary spent in that phase. We evaluate all claims for insulin users (including their non-insulin claims) to identify the date on which they transition into a new benefit phase. For example, if the beneficiary's first claim in the coverage gap occurred on April 1, 2018 (for any drug, whether insulin or not) and first claim in catastrophic coverage occurred on October 1, 2018, they would be assigned 90 days in initial coverage, 183 days in the coverage gap, and 92 days in catastrophic coverage. This provides the denominator for the percent of days covered by benefit phase calculation.

For the numerator, we measure the beneficiary's total basal insulin days supplied while they were in a given benefit phase. We create an indicator for each day in 2018 that is a 1 if the beneficiary possesses unused basal insulin on that day and 0 if they have exhausted their days supplied. If the beneficiary's first basal insulin claim is on January 1, 2018, we start from there, otherwise we start with the date of the beneficiary's last basal insulin claim in 2017 and count forward days supplied from there. For example, if they filled a 30-day basal insulin prescription on December 17, 2017, then we assume they consumed the first 15 days supplied in 2017, leaving them with 15 days supplied to carry forward into 2018. We assign the beneficiary a 1 for January 1 through January 15. If they do not have another insulin fill, then they are assigned a 0 for January 16, and all subsequent days until their next insulin fill. If they have an insulin fill prior to January 16, those days supplied are allocated starting on January 16. For example, if they filled another prescription with 30 days supplied on January 13, 30 days are credited starting January 16 rather than on the 13. That is, we carry forward the days supplied of the new fill until the days supplied of the previous fill are fully exhausted. If the beneficiary takes more than one basal insulin at the same time or switches products mid-year, we calculate these measures in the same way.

© 2021 Trish et al. *JAMA Network Open.*

Finally, we calculate the percent of days covered, by benefit phase, as the percent of days that the beneficiary spent in that benefit phase where they possessed unused days supplied (i.e., where this indicator is a 1).

One limitation of our analysis is that using claims-based measures of days supplied may lead to mismeasurement of insulin adherence. [1] Thus, our measures of the *absolute* level of percent of days covered should be interpreted with caution. However, such mismeasurement would need to occur differentially across phases or by enrollees in employer vs. non-employer plans to affect our measurement of the *changes* in insulin adherence associated with changes in cost-sharing.

eReferences

1. Stolpe, S., et al., A Systematic Review of Insulin Adherence Measures in Patients with Diabetes. ***Journal of Managed Care & Specialty Pharmacy***, 22(11):1224-1246; 2016.

© 2021 Trish et al. *JAMA Network Open.*
